# miR-122 Regulates p53/Akt Signalling and the Chemotherapy-Induced Apoptosis in Cutaneous T-Cell Lymphoma

**DOI:** 10.1371/journal.pone.0029541

**Published:** 2012-01-03

**Authors:** Valentina Manfè, Edyta Biskup, Anne Rosbjerg, Maria Kamstrup, Anne Guldhammer Skov, Catharina Margrethe Lerche, Britt Thyssing Lauenborg, Niels Ødum, Robert Gniadecki

**Affiliations:** 1 Department of Dermatology, Bispebjerg Hospital, Copenhagen, Denmark; 2 Institute of Medical Microbiology and Immunology, University of Copenhagen, Copenhagen, Denmark; 3 Faculty of Health Sciences, University of Copenhagen, Copenhagen, Denmark; Institut Jacques Monod, France

## Abstract

Advanced cutaneous T-cell lymphoma (CTCL) is resistant to chemotherapy and presents a major area of medical need. In view of the known role of microRNAs (miRNAs) in the regulation of cellular signalling, we aimed to identify the functionally important miRNA species, which regulate apoptosis in CTCL. Using a recently established model in which apoptosis of CTCL cell lines is induced by Notch-1 inhibition by γ-secretase inhibitors (GSIs), we found that miR-122 was significantly increased in the apoptotic cells. miR-122 up-regulation was not specific for GSI-1 but was also seen during apoptosis induced by chemotherapies including doxorubicin and proteasome blockers (bortezomib, MG132). miR-122 was not expressed in quiescent T-cells, but was detectable in CTCL: in lesional skin in mycosis fungoides and in Sézary cells purified from peripheral blood. *In situ* hybridization results showed that miR-122 was expressed in the malignant T-cell infiltrate and increased in the advanced stage mycosis fungoides. Surprisingly, miR-122 overexpression decreased the sensitivity to the chemotherapy-induced apoptosis *via* a signaling circuit involving the activation of Akt and inhibition of p53. We have also shown that induction of miR-122 occurred *via* p53 and that p53 post-transcriptionally up-regulated miR-122. miR-122 is thus an amplifier of the antiapoptotic Akt/p53 circuit and it is conceivable that a pharmacological intervention in this pathway may provide basis for novel therapies for CTCL.

## Introduction

Mycosis fungoides (MF) and Sézary syndrome (SS) are the commonest cutaneous T-cell lymphoma (CTCL) [1]. SS is a lymphoma-leukaemia syndrome characterized by erythroderma and the presence of a malignant T-cell clone in the peripheral blood and the skin. Lymphomatous infiltrations in the skin in MF present initially as patches and plaques that eventually progress to tumours with subsequent involvement of lymph nodes and visceral organs [1], [2]. Advanced cutaneous T-cell lymphoma is an incurable disease and represents an area of a high medical need. Classic anthracycline- or nucleoside analog-based regimens provide only short-lived responses and relapses are invariably observed within a year [2], [3]. It has been hypothesized that relapses are caused by a subpopulation of long-lived, mitotically quiescent malignant cells that survive even intensive chemotherapy regimes [4]. Current research efforts are therefore concentrated on a better understanding of chemotherapy resistance in CTCL and on identification of new pharmacological targets [3], [5].

Notch-1 is an evolutionarily conserved receptor that is indispensable for the normal T-cell development [6]–[8]. General mechanisms involved in Notch-dependent leukemogenesis have originally been described in acute lymphoblastic T-cell leukaemia (T-ALL) and include the chromosomal translocation (t (7; 9)) [9] and gain-of function mutations of Notch-1 [10]. Subsequently, Notch has been connected with the pathogenesis of several solid tumours and haematological malignancies [11]. In contrast to the situation seen in T-ALL, the hyperactivation of Notch-1 in these neoplasms is not usually mutation-driven but depends on an excessive proteolytic cleavage by the γ-secretase complex [12] that leads to translocation of the biologically active Notch fragment into the nucleus. Inhibition of Notch can therefore be achieved by γ-secretase inhibitors (GSIs) and GSI compounds advanced to phase I clinical trials for refractory T-ALL (www.clinicaltrials.gov/ct2/show/NCT00100152), breast cancer (www.clinicaltrials.gov/ct2/show/NCT00106145, www.clinicaltrials.gov/ct2/show/NCT00645333) and other solid tumours [13]. Recently, we have provided evidence that Notch-1 is a promising therapeutic target in CTCL. Notch-1 is expressed in MF, SS, and in CD30^+^ cutaneous lymphomas and Notch blockade by GSIs causes apoptosis in various lymphoma cell lines [14], [15].

The major concern associated with the clinical use of GSIs is a significant organ toxicity at therapeutic doses [16]. We have therefore studied the mechanisms determining resistance to chemotherapy in CTCL with a particular focus on microRNA (miRNA)-mediated regulation. miRNAs are short (−22 nt.), highly conserved, noncoding RNAs that regulate gene expression by targeting mRNAs at the 3′ untranslated regions (UTRs) [17], [18]. It is postulated that each miRNA regulates up to 100 different mRNAs and that more than 10,000 mRNAs appear to be directly regulated by miRNAs [19]. These targeted genes control fundamental cellular processes such as cell proliferation and apoptosis [20] and therefore it is not surprising that aberrant miRNA signalling is associated with cancer initiation and progression [21]. We have recently investigated the changes in miRNA expression induced by GSIs and found that several miRNA species are deregulated in CTCL cell lines [22]. Among these, we identified miR-122, which has been considered to be specifically expressed in liver and involved in hepatocarcinogenesis [23], [24]. Since miR-122 has been shown to stimulate hepatocyte apoptosis, we hypothesized that it plays a similar role in lymphoma cells and possibly mediates the pro-apoptotic activity of GSI. Surprisingly, we found that the function of miR-122 is highly tissue specific and that in malignant T-lymphocytes it blocks apoptosis. We describe here a negative regulatory loop in which miR-122 is induced by chemotherapeutic agents *via* p53 and increases cell resistance to apoptosis *via* stimulation of Akt kinase.

## Materials and Methods

### Ethic Statement

The study was approved by the Ethics Committee of Copenhagen and Frederiksberg. Skin biopsies were collected from 21 patients with MF after written informed consent.

### Cell culture and tissue procurement

Three CTCL cell lines have been used: MyLa2000 derived from a plaque biopsy of a patient with MF [25], SeAx [26] and Hut-78 [27] derived from peripheral blood of patients with Sézary syndrome. MyLa2000 and SeAx cells were cultured in DMEM containing 4.5 g/l glucose, 10% fetal bovine serum (FBS) and at 37°C under 5% CO_2_. Hut-78 cells were cultured in RPMI 1640 containing 2 mM L-glutamine and 10% FBS. Human primary T-cells (CD4^+^ and CD8^+^, quiescent T-cells) from three healthy donors were purified using density gradient centrifugation (Ficoll-PlaqueTM PLUS, Amersham Biosciences, Uppsala, Sweden) as previously described [15]. Sézary cells (CD4^+^CD7^−^) were isolated from the peripheral blood of two patients (2 males; age of 56 and 65 years; CD4^+^/CD8^+^ ratio of 19 and 29) using anti-CD7 biotin conjugated antibody (eBioscence, San Diego, CA) and anti-CD4 micro beads (Milteny Biotec) together with the anti-Biotin Multisort Kit (Milteny Biotec, Bergisch Gladbach, Germany), as described by Marzec *et al.* [28]. Lesional skin comprising 10 human punch biopsies were collected from patients with MF (6 males and 4 females; mean age 75 years; range 65 to 90 years; 7 plaque/patch (T2) and 3 tumour (T3) stadium) and stored in RNA*later* (Ambion, Austin TX) before proceeding to tissue homogenization. *In situ* hybridization (ISH) was performed on paraffin sections of 11 patients with MF (7 males and 4 females; mean age 70 years; range 49 to 88 years; 6 plaque/patch (T2) and 5 tumour (T3) stadium). Patient diagnoses were confirmed by an expert pathologist in accordance with the WHO-EORTC classification [1], [29].

### Reagents

GSI-1 (Z-Leu-Leu-Nle-CHO), Ly294002 (2-(4-morpholino)-8-phenyl-4H-1-benzopyran-4-one), Akt inhibitor X (10-(4′-(N-diethylamino) butyl)-2-chlorophenoxazine, HCl) were from Merck Calbiochem (Darmstadt, Germany). Nutlin-3a was from Cayman Chemical (Ann Arbor, MI). Bortezomib (MG341) and MG132 (Z-Leu-Leu-Leu-CHO) were purchased from Selleck (West Paterson, NJ) and doxorubicin hydrochloride from Sigma (Aldrich, St. Louis, MO). Drugs were dissolved in dimethyl sulphoxide (DMSO). Mock-treated cells were cultured with DMSO at final concentrations of 0.04–0.2%.

### RNA purification

Tissue samples were homogenized using a TissueLyzer II (Qiagen, Valencia, CA, USA) and processed as previously described [30]. Briefly, the cells were centrifuged at 600 rpm for 6 minutes and the pellet was washed twice in PBS. Total RNA (TRNA) and small RNAs were purified using the Rneasy® MiniKit (Qiagen, Hilden, Germany) and mirVana MicroRNA Isolation Kit (Ambion, Foster City, CA) according to the manufacturer's instructions. The concentration of TRNA was measured spectrophotometrically using NanoDrop ND-1000 (Thermo Scientific, Wilmington, DE), and RNA integrity was confirmed with Agilent 2100 Bioanalyzer using Agilent Nano RNA kit (Agilent Technologies, Santa Clara, CA). High quality total RNA from human liver was from Ambion.

### Quantification of miRNA expression by real-time q-PCR

Mature miRNA expression was measured in the samples containing the same amount of TRNA and small RNAs with quantitative real-time qRT-PCR assay (TaqMan™ microRNA Reverse Transcription kit-4366596, and TaqMan™ Universal PCR Master mix-4324018, Applied Biosystems, Foster City, CA) according to the manufacturer's instructions. miR-191 was used as a reference for the normalization of RT-PCR-data [31].

For detection of pri-miR-122, cDNA was prepared from 1 µg of TRNA per reaction using High Capacity cDNA Reverse Transcription Kit (Applied Biosystems) and amplified using TaqMan® Pri-miRNA Assay and TaqMan® Gene Expression Master Mix mix-4324018 (Applied Biosystems) according to the manufacturer's instructions. 18S rRNA was used as an internal control gene.

The RT-PCR was performed in triplicates using a 7900HT Fast Real-Time PCR System (Applied Biosystems).

### *In situ* hybridization

ISH was done on skin lesions of patients with MF as previously described [32]. Paraffin sections were mounted on SuperFrost®Plus slides (Dako, Glostrup, Denmark), air-dried for 1–2 h at RT, melted in the oven at 60°C for 45 min, deparaffinized in xylene and hydrated through decreasing ethanol concentrations into PBS. Sections were then treated by proteinase-K (15 µg/ml in PK-buffer, 5 mM Tris-HCl, pH 7.4, 1 mM EDTA, 1 mM NaCl) at 37°C for 8 min in a volume of 300 µL in a Dako hybridizer (Dako), washed, and dehydrated through an increasing gradient of ethanol solutions. Two different mercury locked nucleic acid (LNA) miRNA Detection Probes (Exiqon, Vedbaek, Denmark) were used; hsa-miR-122 (40 nM in a formamide-free ISH buffer) and scrambled probe (40 nM). Probes were denatured by heating to 90°C for 4 min and 50 µL probe mixture was hybridized with the tissue sections in the hybridizer at 55°C for 60 min. The slides were then placed at RT in 5× saline-sodium citrate (SSC) (Invitrogen) and washed for 5 min at 55°C in 5× SSC (one wash), 1× SSC (two washes) and 0.2× SSC (two washes). After a further washing in PBS, sections were blocked with DIG Wash and Block Buffer Set (Roche, Mannheim, Germany) and incubated for 60 min at RT with alkaline phosphatase (AP)-conjugated anti-DIG (diluted 1∶800 in blocking solution, Roche). Slides were then washed twice with PBS containing 0.1% Tween-20. Ready to use tablets (Roche) of 4-nitro-blue tetrazolium (NBT) and 5-brom-4-chloro-3′-indolylphosphate (BCIP) substrate were dissolved in aqueous 0.2 mM levamisole. Slides were incubated for 120 min at 30°C to develop the dark-blue NBT-formazan precipitate, washed twice for 5 min in KTBT buffer (50 mM Tris–HCl, 150 mM NaCl, 10 mM KCl), and then twice in water, dehydrated in the ethanol gradient and mounted.

Skin sections were classified into three categories depending on the percentages (<25%, 25–75%, >75%) of malignant T-cells positive for miR-122 staining.

### siRNA and miRNA Transfection

In the case of SeAx cells, transfection was carried out using Lipofectamine RNAiMax (Invitrogen, Carlsbad, CA) according to the manufacturer's protocol as previously described by [15]. 20 nM miRIDIAN miRNA Mimics and Hairpin Inhibitors from Dharmacon (Thermo Scientific, Chicago, Il) were used for specific inhibition and overexpression of miR-122. Small Interfering RNA was used at 50 nM for the specific knockdown of PTEN (6251, Cell Signalling, Beverly, MA) and p53 (6231, Cell Signalling).

In the case of MyLa2000 cells, transfection was carried out using 4 µl TurboFect™ *in vitro* Transfection Reagent (Fermentas, Burlington, Canada). 50 nM miRIDIAN miRNA Mimics and Hairpin Inhibitors from Dharmacon (Thermo Scientific, Chicago, Il) were mixed to 200 µl GIBCO™ Opti-MEM I (Invitrogen, Carlsbad, CA) and incubated for 20 min before application to 500.000 cells/mL.

Scrambled siRNA (Dharmacon) was used as a negative control. Efficiency of transfection was evaluated by SiGLO (Dharmacon) and by RT-PCR (Figure S1).

### Apoptosis and cell viability

Apoptosis was determined by flow cytometry using FITC-annexin V/propidium iodide (PI) protocol of the manufacturer (Beckman Coulter), as previously described [15]. In some experiments the proportion of viable cells was determined by staining with only PI (5 µg/ml).

### Cells extracts and western blotting

Whole cell extracts were prepared as described previously [15] and equal amounts of protein were separated by a 12% Bis-Tris gel electrophoresis at 200 V followed by electrophoretic transfer to a nitrocellulose membrane (Bio-Rad Laboratories, Hercules, CA). Membranes were then blocked for 1 hour at 4°C with Li-Cor blocking agent (Lincoln, NE), incubated with primary mouse or rabbit antibody overnight at 4°C followed by incubation for 1 hour with the appropriate secondary antibodies labeled with 800IR dye (anti-rabbit) (Li-Cor) or Alexa Fluor 680 (anti-mouse) (both from Molecular Probes, Invitrogen Cooperation, Carlsbad, CA). The following primary antibodies were used: Akt total, phosphorylated Akt (Ser^473^), total FoxO3a, phosphorylated FoxO3a (Ser^318/321^), p53, phosphorylated MDM2 (Ser^166^), P70 S6K total and phosphorylated P70 S6K (Thr^421/Ser424^) from Cell Signalling (Beverly, MA), anti–β-actin antibody from Sigma (Aldrich, St. Louis, MO). Immunoreactivity was detected and quantified with the infrared Odyssey imaging System (Li-Cor).

### Statistics

Continuous data are reported as means with standard deviation (SD) and the differences were evaluated by the Student's *t*-test. RT-PCR data were analyzed using the ΔΔCT method by paired Student's t-test. Normality of the data was assessed by Kolmogoroff-Smirnov test. Correlation between miR-122 expression level in the MF biopsies and the stage was analyzed with χ^2^ test. *p*-value<0.05 was considered significant. Statistical analysis was performed by the SPSS Version 17.0 (SPSS Inc., Chicago, Il), GraphPad Prism Version 4.03 (GraphPad Software Inc., San Diego, CA) or Excel (Microsoft Corp., Redmond, WA).

## Results

### miR-122 is expressed in CTCL and inhibits apoptosis induced by chemotherapy

**Figure 1:**
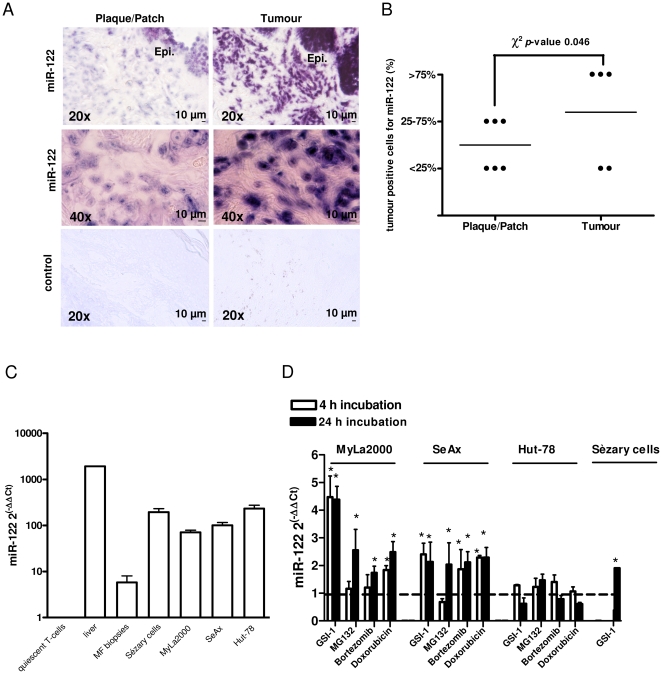
Expression of miR-122 in CTCL. A–B) Analysis of miR-122 expression using ISH and miR-122-specific LNA probe in lesional skin sections from patients with plaque/patch (n = 6) or tumour-stage (n = 5) MF. A) Representative ISH staining using miR-122 or scrambled (control) probes in MF skin sections (plaque/patch-stage MF, left panels; tumour-stage MF, right panels). The blue-purple color indicates miR-122 expression. Magnification: 20× and 40×. Abbreviation: Epi., epidermis. B) Skin biopsies were classified into three categories depending on the percentages of miR-122 positive tumour cells, as described in Methods. Comparison of the miR-122 expression profile showed an increase of miR-122 expression in the tumour-stage MF (*p*-value = 0.046). C) qRT-PCR measurements of miR-122 expression in human liver, in a panel of CTCL cell lines (MyLa2000, SeAx and Hut-78) and *in vivo* in leukaemic cells from two patients with Sézary syndrome (Sézary cells) and in lesional skin from 10 patients with MF. miR-122 expression levels were normalized to the relative expression in quiescent T-cells. *Columns*, mean (n = 3); *bars*, SEM. D) Induction of miR-122 during chemotherapy-induced apoptosis in CTCL cell lines (MyLa2000 SeAx, Hut-78) and in malignant lymphoma cells isolated from peripheral blood of a patient with Sèzary syndrome (Sézary cells). The cells were incubated with 0.8 µM GSI-1, 30 nM MG132, 6 nM bortezomib and 1 µM doxorubicin for 4 and 24 h, as indicated, and miR-122 expression was quantified by qRT-PCR. Because the Sézary cells could not be purified in large quantities, the experiments were only performed with 0.8 µM GSI-1. Data show fold-changes in miR-122 level after treatment as compared to the control, mock-treated cells. *Columns*, mean (n = 3); *bars*, SEM. * *p*-value<0.05 versus untreated cells.

In order to identify the miRNA species involved in the regulation of apoptosis in CTCL, we investigated the influence of GSIs on miRNA expression pattern in CTCL cell lines. The rationale for choosing GSIs was our previous studies showing that these agents potently induce apoptosis *via* the inhibition of the proteolytic cleavage of Notch [8], [14], [15]. Preliminary results obtained from miRNA arrays were described by Manfé *et al.* [22] and revealed miRNA species, differentially expressed between GSI-1 treated and control cells (miRNAs down-regulated: miR-27a, miR-92b, miR-181a, miR-18a, miR-425, miR-193b, miR-30d, miR-199a-5p, miR-19b, miR-222, miR-221, miR-9*, miR-125a-5p, miR-23b; miRNAs up-regulated: miR-122, miR-214, miR-185, miR-574-5p, miR-638, miR-149*, miR-766, miR-595). Subsequent functional analysis using the corresponding antagomiRs and mimic oligonucleotides showed a functional significance of one of the deregulated miRNAs: miR-122. miR-122 has been considered to be liver specific but recent evidence showed that this miRNA is more widely distributed and is also detected in human skin [30]. Here, we analyzed miR-122 expression by LNA-ISH in 11 sections of lesional skin biopsies of patients with MF. Figure 1A shows miR-122 presence in the epidermis and in the infiltrate of malignant T-cells in plaque/patch (T2) and tumour (T3) stage MF. LNA-ISH with a control scrambled RNA did not give any signal (Figure 1A). miR-122 positive signal was significantly increased in the advanced stage MF (χ^2^ test, *p-*value = 0.046); 3 of 5 cases of tumour, but none of the 6 cases of plaque/patch, displayed more than 75% of malignant T-cells positive for miR-122 (Figure 1B). We concluded that miR-122 expression correlated with the T-stage in MF.

We also measured miR-122 expression level *in vitro* and *in vivo* in CTCL by TaqMan real-time RT-PCR. As shown in Figure 1C, miR-122 was not detectable in quiescent-T cells, but it was expressed in three CTCL cell lines (SeAx, MyLa2000, HuT-78), in leukaemic cells purified from the patients with Sézary syndrome and in the lesional skin from patients with MF. However, the miR-122 expression in CTCL was lower than the one in human tissue liver. Exposure to GSI-1 increased the expression of miR-122 in SeAx and MyLa2000 cells as previously reported [22], but not in Hut-78 cells. This effect was not specific to GSI since proteasome inhibitors (another class of chemotherapeutic agents which induce apoptosis in CTCL (15)) and doxorubicin increased miR-122 expression in SeAx and MyLa2000 cells (Figure 1D). The treatment of purified leukaemic Sézary cells with GSI-1 also increased the expression of miR-122 (Figure 1D).

**Figure 2:**
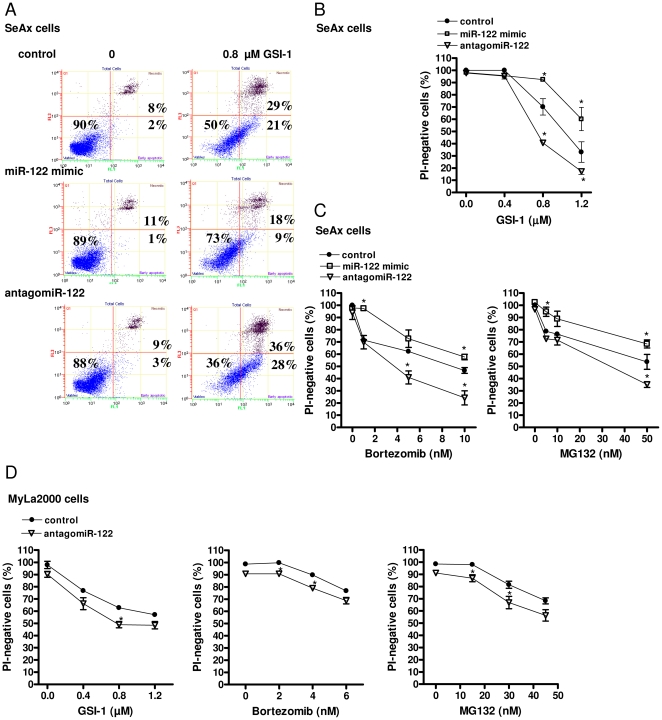
miR-122 increases resistance to GSI-1 and proteasome inhibitors. A) SeAx cells were transfected for 24 h with either scrambled siRNA (control), miR-122 mimic or antagomiR-122 and treated for 48 h with GSI-1 (0.8 µM) followed by annexin V and propidium iodide staining for flow cytometry, as described in Methods. The dot-plot graphs (annexin V: green FL1 channel, x-axis; PI: red FL3 channel, y-axis) are representative of three independent experiments. B–C) Effect of antagomiR-122 and miR-122 mimic on the apoptosis induced by GSI-1 (at the concentrations 0.4, 0.8 and 1.2 µM; 48 h) (B), bortezomib (1, 5, 10 nM; 48 h) (C, left panel) and MG132 (5, 10, 50 nM; 48 h) (C, right panel) in SeAx cells. Cell viability is expressed as percentage of the PI-negative cells calculated by flow cytometry. *Points*, mean (n = 3); *bars*, SEM. *p-value<0.05 compared to cells transfected with scrambled oligonucleotide. D) MyLa2000 cells were transfected for 24 h with either scrambled siRNA (control) or antagomiR-122 and treated for additional 24 h with GSI-1 (at the concentrations 0.4, 0.8 and 1.2 µM) (D, left panel) bortezomib (2, 4, 6 nM) (D, middle panel) and MG132 (15, 30, 45 nM) (D, right panel). The percentage of the PI-negative cells was calculated as in (B). *Points*, mean (n = 3); *bars*, SEM. *p-value<0.05 compared to cells transfected with scrambled oligonucleotide.

We further examined the effect of miR-122 up-regulation on apoptosis in CTCL cell lines. We attenuated miR-122 using miR-122 hairpin inhibitor (antagomiR-122) in GSI-1-treated or untreated SeAx cells (Figure 2A, B). SeAx treatment with antagomiR-122, but not with the scrambled oligonucleotide, led to a substantial increase in apoptosis induced by GSI-1 but did not affect apoptosis in the untreated cells. mir-122 overexpression had an opposite effect and protected against GSI-1 induced apoptosis. We observed similar results with MG132 and bortezomib (Figure 2C). We also examined the impact of attenuation of miR-122 expression on apoptosis in another CTCL cell line, MyLa2000. Similar to SeAx cells, we observed an increase in the number of apoptotic cells after GSI-1, bortezomib and MG132 treatment in miR-122 depleted cells relative to the scrambled oligonucleotide control (Figure 2D). Taken together, our data suggested that miR-122 conferred a protective response of malignant T-cells to chemotherapy-induced apoptosis in CTCL.

### miR-122 protects against apoptosis by activation of Akt signalling

**Figure 3:**
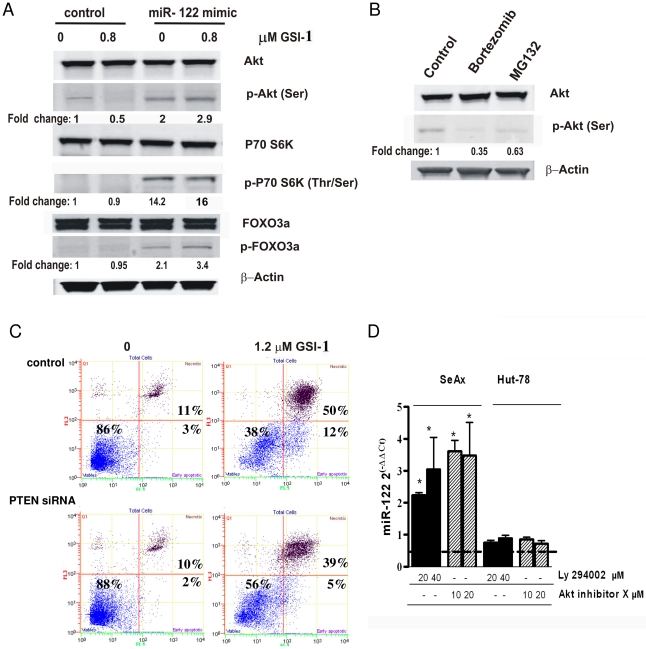
Reciprocal regulation between miR-122 and Akt. A) miR-122 activates Akt signalling in SeAx cells. Immunoblots on whole cell-lysates from scrambled siRNA (control) or miR-122 mimic-transducted cells exposed to GSI-1 (0.8 µM; 4 h) and probed for the indicated proteins of the Akt signalling pathway. ß-actin was used as housekeeping gene, and relative protein expression levels are reported below the corresponding western blot bands. The blots shown are representative from three independent experiments. B) Inhibition of Akt by bortezomib and MG132. SeAx cells were treated with 6 nM bortezomib and 30 nM MG132 for 4 h and Akt activity was assessed in whole cell lysates by western blotting. C) Akt activation protects against GSI-1-induced apoptosis. Decrease in apoptotic, annexin V positive cells in SeAx transfected with PTEN siRNA and exposed to GSI-1 (1.2 µM, 24 h), comparing to the scrambled siRNA (control). Flow cytometry was done as described in Figure 2A. Graphs are representative of three independent experiments. D) Akt inhibition induces miR-122 expression. qRT-PCR measurement of miR-122 level in SeAx and Hut-78 cells treated with the indicated doses of Ly294002 and Akt inhibitor X for 24 h and expressed as fold change compared to those in the untreated cells. *Columns*, mean (n = 3); *bars*, SEM. * *p*-value<0.05 versus untreated cells.

Our next aim was to determine which signalling pathways involved in apoptosis are modulated by miR-122. It is known that apoptosis in SeAx cells can be caused by the inhibition of Stat3 or Akt signalling [8], [33], [34]. Overexpression of miR-122 did not inhibit either Stat3 or NF-kB (data not shown). However, we found that miR-122 overexpression induced Akt phosphorylation at Ser^473^ after 4 hours (∼2 fold increase compared with control siRNA) (Figure 3A). We confirmed the functional activation of Akt in miR-122 overexpressing cells by showing that the downstream targets of Akt, p70 S6K and Forkhead FOXO3a transcription factor (FXHRL1), were phosphorylated (Figure 3A). The pro-apoptotic drugs (GSI-1, bortezomib and MG132) decreased the Akt phosphorylation (Figure 3A, B) and the simultaneous treatment with miR-122 mimic and GSI-1 recovered Akt activity (Figure 3A). These data indicated that miR-122 inhibited apoptosis *via* activation of Akt. We further confirmed that Akt activation was functional in the regulation of apoptosis by showing that inhibition of PTEN, the major negative regulator of Akt, protected the SeAx cells from GSI-1-induced cytotoxicity (Figure 3C).

Interestingly, we observed a reciprocal regulation between miR-122 and Akt. In addition to the above described activation of Akt by miR-122, the inhibition of Akt activity by 2 different blockers, Ly294002 (a potent inhibitor of phosphoinositide 3-kinases, PI3Ks) and Akt Inhibitor X (inhibitor of the phosphorylation of Akt and its *in vitro* kinase activity) induced expression of miR-122 in SeAx cells (Figure 3D). Notably, no miR-122 increase following the Akt inhibition was observed in Hut-78 cells (Figure 3D).

### miR-122 induction depends on p53

**Figure 4:**
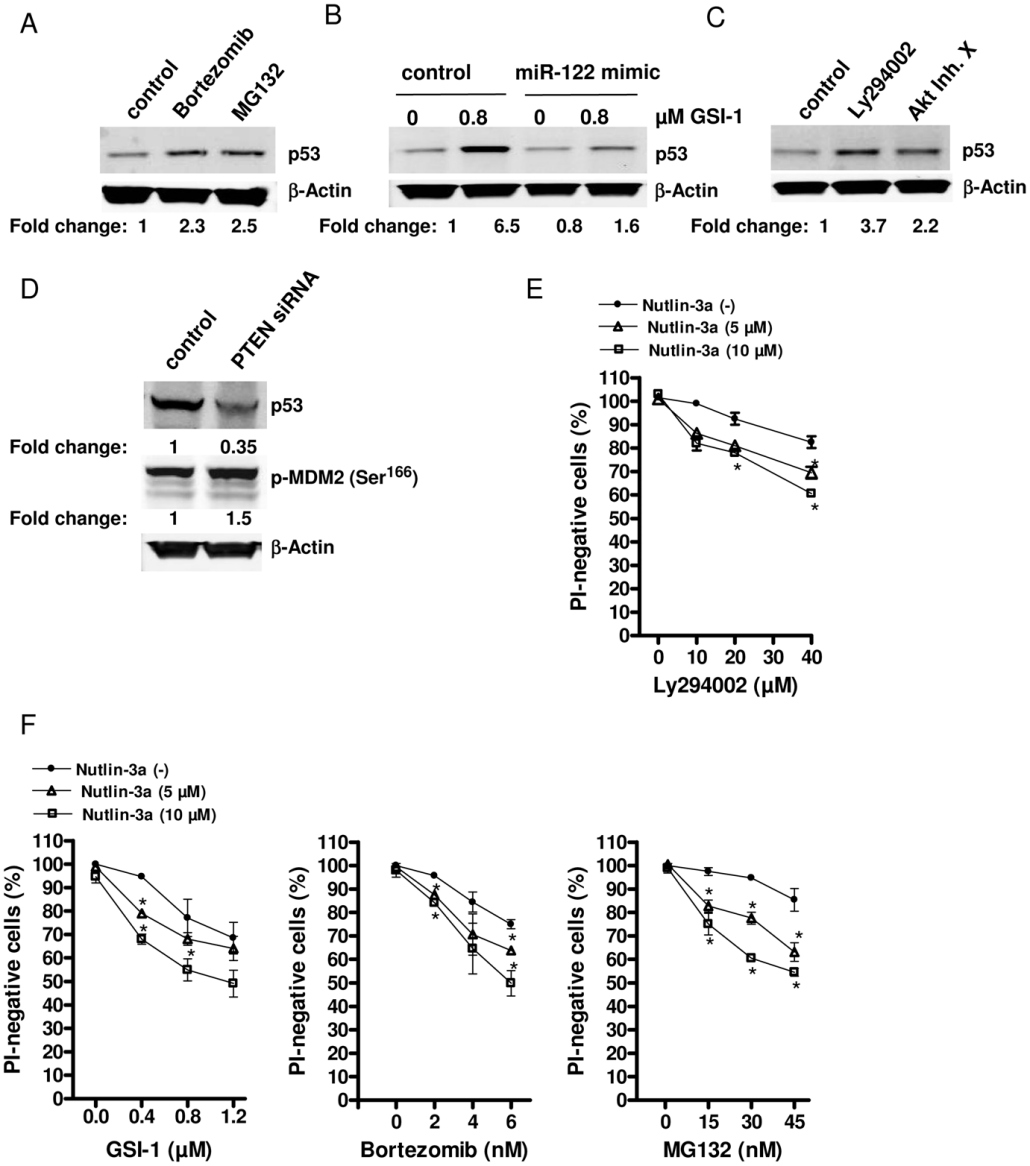
p53 amplifies the chemotherapy-induced apoptosis. A) p53 protein level was assessed by western blot analysis in SeAx cells treated for 4 h with 6 nM bortezomib or 30 nM MG132. ß-actin was used as housekeeping gene, and relative p53 expression is reported as in Figure 3. B) miR-122 mimic counteracts the GSI-1-induced p53 activation. Cells transfected with either a control siRNA or miR-122 mimic for 24 h were exposed to GSI-1 for 4 h, at indicated concentrations. p53 expression was determined by western blot as in (A). C–D) Regulation of p53 by Akt. SeAx cells were treated for 24 h with 20 µM Ly294002 or 10 µM Akt Inhibitor X (C) or transfected with PTEN or the scrambled, control siRNA (D). p53 was measured as in (A). E–F) p53 activation by nutlin-3a increases the chemotherapy-induced apoptosis. SeAx were exposed to 5 or 10 µM nutlin-3a for 4 h followed by 20 h treatment with Ly294002 (at the concentrations 10, 20 and 40 µM) (E) or GSI-1 (0.4, 0.8, 1.2 µM) (F, left panel)), bortezomib (2, 4, 6 nM) (F, middle panel), MG132 (15, 30, 45 nM) (F, right panel). Cell viability was determined as in Figure 2. *Points*, mean (n = 3); *bars*, SEM. * *p*-value<0.05, versus untreated cells.

To elucidate whether the observed increase in miR-122 is a direct consequence of Akt inhibition or is mediated by another protein we focused on a possible role of p53. The idea that p53 might be involved arose from the observations in another T-cell neoplasm, T-ALL, where hyperactivation of Notch-1 inhibits PTEN, which in turn leads to activation of Akt and p53 degradation *via* induction of human homolog of the mouse double minute 2 p53-binding protein (MDM2) (for a review see Dotto [35]). We observed that GSI-1, bortezomib and MG132 induced p53 accumulation in SeAx cells (Figure 4A, B) and that miR-122 mimic oligonucleotide inhibited this response (Figure 4B). Third, Akt inhibition by Ly294002 and Akt Inhibitor X increased p53 (Figure 4C) and the activation of Akt by siRNA against PTEN increased the phosphorylation of Ser^166^ on MDM2 and caused a degradation of p53 (Figure 4D).

**Figure 5:**
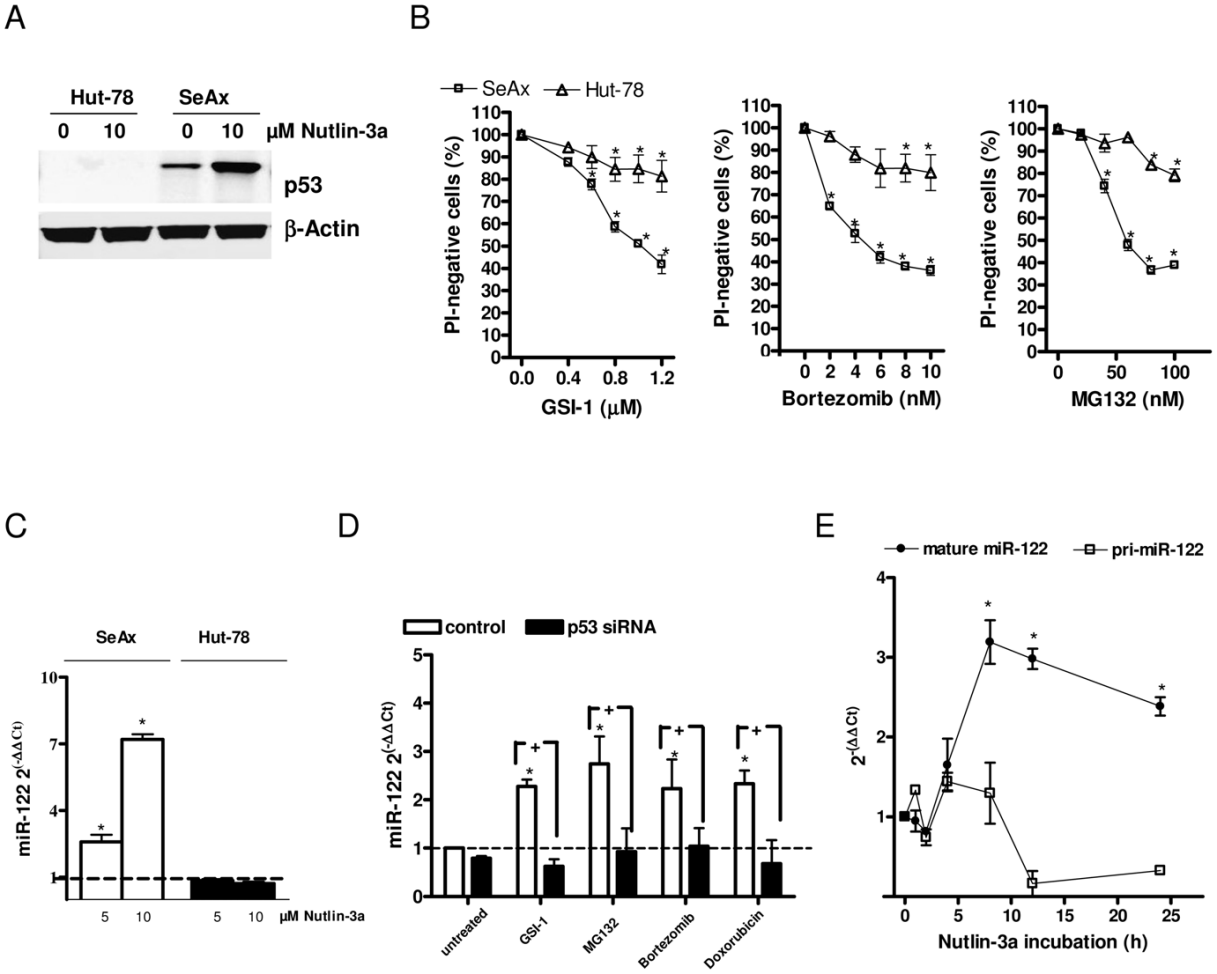
miR-122 induction depends on p53. A) p53 immunoblots of whole-cell lysates from Hut-78 (*P53* ^mut^) and SeAx cells treated with nutlin-3a (10 µM; 24 h). B) Dose-dependent effect of GSI-1 (at the concentrations 0.4, 0.6, 0.8, 1 and 1.2 µM), bortezomib (2, 4, 6, 8, 10 nM) and MG132 (20, 40, 60, 80, 100 nM) on apoptosis in SeAx and Hut-78 cell lines. Cell viability was determined as in Figure 2B. *Points*, mean (n = 3); *bars*, SEM. * *p*-value<0.05 versus untreated cells. C) Quantification of miR-122 expression by qRT-PCR in SeAx and Hut-78 cells treated with the indicated doses of nutlin-3a for 24 h. miR-122 levels after treatment are expressed as fold change compared to the mock-treated cells as in Figure 1D. *Columns*, mean (n = 3); *bars*, SEM. * *p*-value<0.05, versus untreated cells. D) p53 siRNA counteracts the miR-122 induction in SeAx cells. qRT-PCR measurement of miR- 122 level in SeAx cells transfected with p53 siRNA or scrambled oligonucleotide and treated with 0.8 µM GSI-1, 30 nM MG132, 6 nM bortezomib and 1 µM doxorubicin for 24 h. Data show fold-changes in miR-122 level after treatment as compared to the control, mock-treated cells. Columns, mean (n = 3); bars, SEM. * *p*-value<0.05 versus untreated cells, ^+^ *p*-value<0.05 versus cells transfected with scrambled oligonucleotide. E) SeAx cells were sampled at various time points during the exposure to 5 µM nutlin-3a and the expression of the mature miR-122 or miR-122 primary transcript (pri-miR-122) was quantified by qRT-PCR. *Points*, mean (n = 3); *bars*, SEM. * *p*-value<0.05, versus untreated cells.

To further investigate the role of p53 in apoptosis in CTCL we used nutlin-3a that is a known inducer of p53 acting *via* disruption of p53-MDM2 interaction [36]. Nutlin-3a amplified the apoptosis induced by Akt inhibition (Figure 4E) and was also able to sensitize SeAx cells to GSI-1, MG132 and bortezomib (Figure 4F). However, the up-regulation of p53 by nutlin-3a did not change the phosphorylation status of Akt (Figure S2A) and did not cause apoptosis (Figure S2B). The down-regulation of p53 by a specific siRNA (Figure S2C) did not affect Akt activation either (Figure S2D). Thus, p53 modulated the sensitivity of SeAx cells to apoptosis, but did not cause cell death on its own. This conclusion was further reinforced by analysis of another CTCL cell line, Hut-78. These cells have a mutated *P*5*3* (*P53* ^mut^) [37] and did not accumulate p53 after nutlin-3a treatment (Figure 5A) and were more resistant than SeAx to the apoptosis after treatment with GSI-1, bortezomib and MG132 (Figure 5B).

The induction of miR-122 during apoptosis seemed to occur *via* p53. In SeAx cells nutlin-3a led to p53 accumulation (Figure 5A) and potently induced miR-122 (Figure 5C). In contrast, in Hut-78 cells, harbouring a truncated form of p53 [37], we observed neither a stabilization of p53 nor up-regulation of miR-122 after exposure to nutlin-3a (Figure 5A, C). Similarly, GSI-1, bortezomib and MG132 induced p53 (Figure 4A, B) and miR-122 in SeAx cells, but not in Hut-78 cells (Figure 1D). Therefore, we determined whether the miR-122 up-regulation by chemotherapies was mediated by p53. p53 in SeAx cells was down-regulated with siRNA and the cells were exposed for 24 h to GSI-1, bortezomib, MG132 and doxorubicin. The expression of miR-122 was significantly up-regulated after the treatment in the control cells transfected with scrambled oligonucleotide but not in the cells in which p53 was silenced by siRNA (Figure 5D). In order to examine whether the miR-122 modulation by p53 in SeAx cells was mediated transcriptionally or post-transcriptionally, we used qRT-PCR for examining changes in miR-122 primary transcript (pri-miR-122) and mature form (mature miR-122) levels following nutlin-3a exposure (Figure 5E). Time course analysis revealed no significant change in pri-miR-122 (less than two-fold), but showed an increased mature miR-122 expression upon nutlin-treatment. Thus, we suggest that p53 modulation of miR-122 occurs post-transcriptionally.

**Figure 6:**
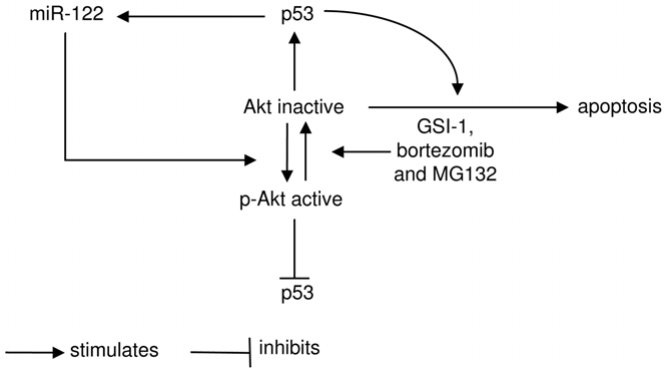
A model of miR-122 action. Chemotherapy, such as GSI-1 or proteasome blockade (bortezomib and MG132) leads to inactivation of Akt and further to apoptosis. Akt inactivation induces p53, which further amplifies Akt-dependent apoptosis, but is unable to induce apoptosis by itself. Another function of p53 is to activate miR-122, which constitutes a cytoprotective signalling loop *via* activation of Akt, and decrease in p53.

The results of our experiments led to a model of miR-122 action outlined in Figure 6. Akt inactivation is a major effector pathway in drug-induced apoptosis and cell death is additionally amplified by p53. On the other hand, the p53 signalling leads to the induction of miR-122, which is antiapoptotic *via* activation of Akt.

## Discussion

This study shows for the first time that miR-122, previously thought to be specific for liver, is expressed *in vivo* in lymphoma cells in CTCL and is further induced during chemotherapy-induced apoptosis. miR-122 expression is up-regulated in advanced stage MF, which may be clinically relevant since the tumour stage (T3) is associated with poor prognosis compared to T2. In contrast to what has been observed in the liver, miR-122 protects against chemotherapy-induced cytotoxicity. The likely mechanism by which miR-122 exerts its cytoprotective effect in lymphoma cells involves p53 and Akt (Figure 6).

The Akt signalling is an important survival pathway essential for the maintenance of malignant cell clones in different cancers, including CTCL [33], [38]. We have advanced the understanding of the role of Akt in CTCL by showing an interplay between Akt and p53. Akt inactivation induced p53 that amplified the pro-apoptotic signal. However, p53 induction did neither provide a sufficient signal for apoptosis nor could it change the phosphorylation status of Akt in SeAx cells. A similar Akt-p53 regulatory circuit has been described in T-ALL [35], [39], [40], and in other neoplasms as cervival carcinogenesis [41]. We suggest that the *P53* status might be an important determinant of treatment outcomes for the lymphoma chemotherapies that predominantly act *via* the Akt pathway. Interestingly, *P53* mutations are rare in cutaneous lymphomas, and are found in very late stages during disease progression [42] and p53 overexpression is associated with large-cell transformation even in the absence of gene mutations [43].

miR-122 is a potent regulator of this pathway. Akt inactivation, either directly by chemical antagonist (Akt Inhibitor X), by PI3K blockers (Ly294002), or indirectly by chemotherapeutic agents induced miR-122 expression. In turn, overexpression of miR-122 activated Akt and increased the resistance of the cells to apoptosis. The detailed mechanism of miR-122 action remains unknown. miR-122 affected the phosphorylation status of Akt but not the amount of Akt protein, arguing against the possibility of direct targeting. We could not detect any effect of miR-122 on the essential upstream Akt kinase, PTEN, the regulatory subunit of PI3K (p85a) or protein phosphatases such as PH domain leucine-rich repeat protein phosphatase (PHLPP) (data not shown). The latter was especially relevant, since the isoform PHLPP2 has been predicted as a direct target of miR-122 using the computational prediction tool miRanda (microRNA.org). However, we detected an effect of miR-122 on a known direct target, cyclin G1 [24]. The role of cyclin G1 is controversial, since it was shown to act as oncogene [24], [44] or tumour suppresor gene [45], [46], depending on cellular context and different experimental settings. According to previous reports [24], a 1.7-fold decrease of cyclin G1 was observed after miR-122 overexpression (data not shown).

The miR-122 induction depended on the presence of p53, as chemotherapies failed to induce miR- 122 expression in *P53* ^mut^ Hut-78 cells and specific attenuation of endogenous p53 with siRNA counteracted the miR-122 up-regulation by chemotherapeutic agents in SeAx cells. However, the presence of high baseline levels of miR-122 in Hut-78 cells suggests the existence of p53-independent pathways of miR-122 regulation. These pathways were not activated by the anticancer drugs tested in this study.

Our results suggest that targeting of miR-122 may improve the outcome of chemotherapy in CTCL. miRNA-based therapy is a rapidly developing field and this approach seems to be feasible in the treatment of cancer [47], [48]. Interestingly, the LNA-antimiR-122 has been developed for the treatment of hepatitis C and has shown efficacy after systemic administration in non-human primates (Miravirsen, Santaris Pharma A/S) [49]–[51]. Miravirsen has currently progressed into phase 2 clinical development to treat patients with chronic hepatitis C. Our work provides a rationale for the use of this drug as a chemotherapy sensitizer in therapy-resistant CTCL.

## Supporting Information

Figure S1 **Efficiency of miR-122 transfection evaluated by quantitative RT-PCR.** A-B) miR-122 level was quantified in SeAx (A) or MyLa2000 cells (B) transfected with scrambled (control), miR-122 mimic or antagomiR-122 oligonucleutides and then exposed to 0.8 µM GSI-1 for 24 h. miR-122 expression is normalized to the relative miR-122 level in the control sample. *Columns*, mean (n = 3).
(TIF)

Figure S2 **Effect of p53 deregulation on Akt.** A) Akt activity in SeAx cells exposed to nutlin-3a (10 µM; 24 h) assessed by western blotting. Relative expression of phosphorylated Akt is reported as fold change using ß-actin as a housekeeping gene. Representative of three independent experiments. B) Cell viability, expressed as percentage of the PI-negative cells, was assessed in SeAx cells exposed to nutlin-3a (2.5, 5, 10, 20 µM; 24) by flow cytometry. *Points*, mean (n = 3); *bars*, SEM. C) SeAx cells were transfected with either scrambled (control) or p53 siRNA and then exposed to nutlin-3a (10 µM) for 24 h. To measure p53 protein level the cells were fixed in 70% ethanol overnight, permeabilized with Triton X-100 and incubated with primary antibody against p53 for 1 h at 37°C. p53 detection was assessed by a secondary Alexa fluor 488-coniugated antibody. Cellular DNA was stained with 7-amino-actinomycin D (7AAD; Beckman Coulter, Fullerton, CA) and the amount of p53 was determined by flow cytometry in a Cell Lab Quanta SC MPL flow cytometer. Nutlin-3a increased p53 amount in the control sample but not in p53 siRNA-transfected cells. D) Immunoblots on whole cell-lysates from scrambled (control) or p53 siRNA-transfected cells probed for the Akt phosphorylation status.
(TIF)
