# Supplementary figures and images for: miR-122 Regulates p53/Akt Signalling and the Chemotherapy-Induced Apoptosis in Cutaneous T-Cell Lymphoma
Source: PLoS One. 2012 Jan 3;7(1):e29541. doi: 10.1371/journal.pone.0029541 (PMC3250447; doi:10.1371/journal.pone.0029541)

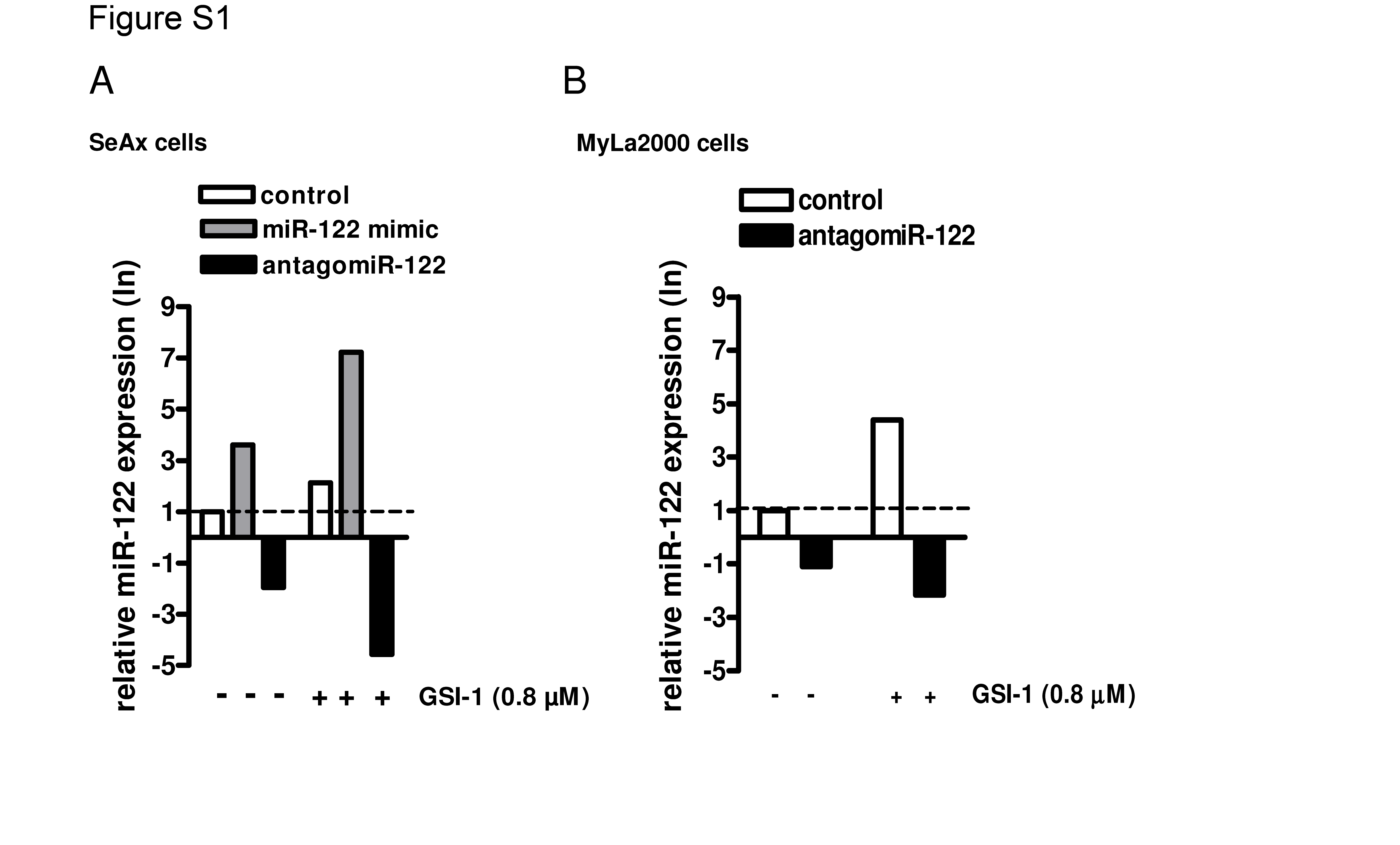

Supplement: Figure S1 — Efficiency of miR-122 transfection evaluated by quantitative RT-PCR. A-B) miR-122 level was quantified in SeAx (A) or MyLa2000 cells (B) transfected with scrambled (control), miR-122 mimic or antagomiR-122 oligonucleutides and then exposed to 0.8 µM GSI-1 for 24 h. miR-122 expression is normalized to the relative miR-122 level in the control sample. Columns, mean (n = 3). (TIF) [file pone.0029541.s001.tif]

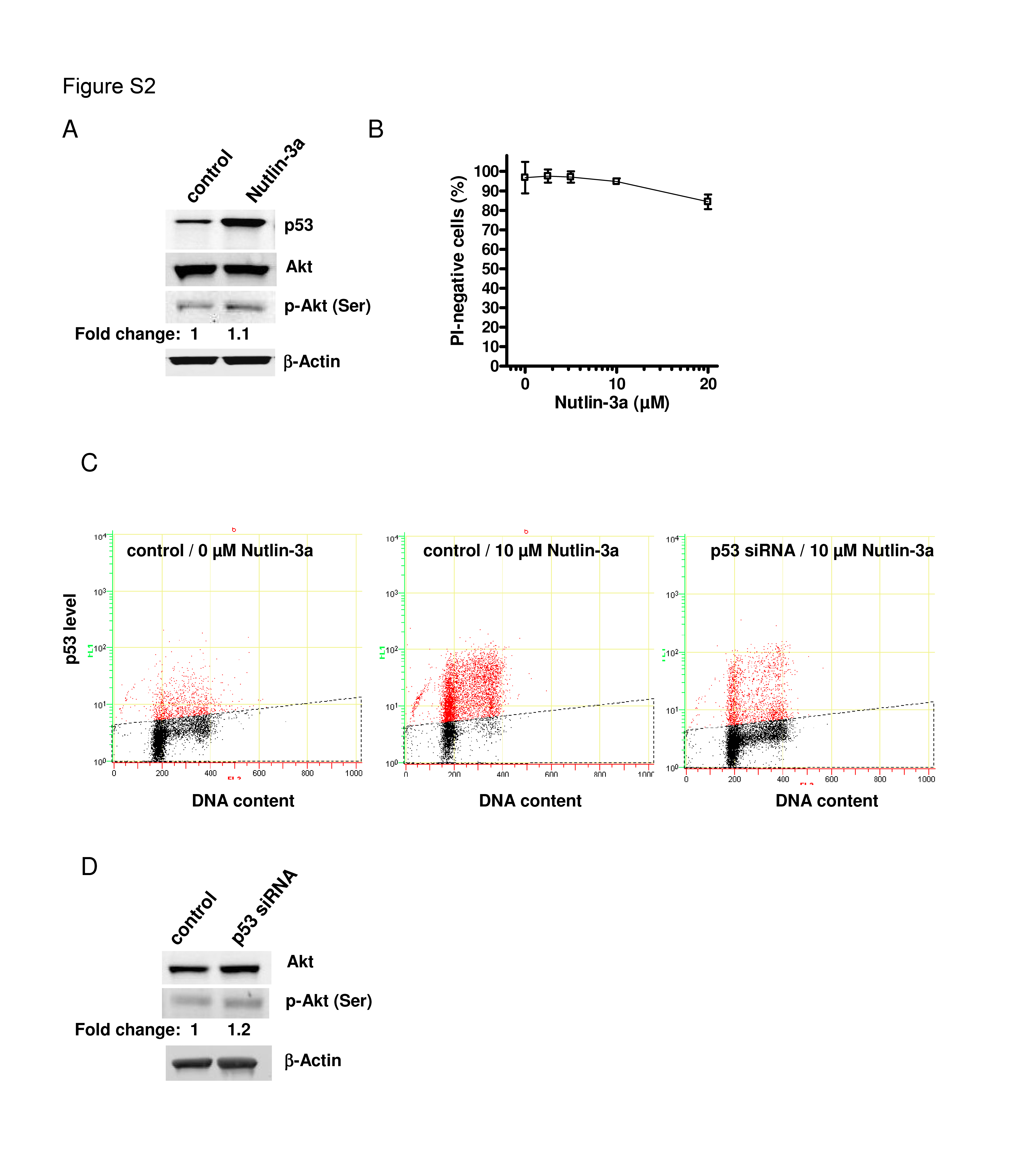

Supplement: Figure S2 — Effect of p53 deregulation on Akt. A) Akt activity in SeAx cells exposed to nutlin-3a (10 µM; 24 h) assessed by western blotting. Relative expression of phosphorylated Akt is reported as fold change using ß-actin as a housekeeping gene. Representative of three independent experiments. B) Cell viability, expressed as percentage of the PI-negative cells, was assessed in SeAx cells exposed to nutlin-3a (2.5, 5, 10, 20 µM; 24) by flow cytometry. Points, mean (n = 3); bars, SEM. C) SeAx cells were transfected with either scrambled (control) or p53 siRNA and then exposed to nutlin-3a (10 µM) for 24 h. To measure p53 protein level the cells were fixed in 70% ethanol overnight, permeabilized with Triton X-100 and incubated with primary antibody against p53 for 1 h at 37°C. p53 detection was assessed by a secondary Alexa fluor 488-coniugated antibody. Cellular DNA was stained with 7-amino-actinomycin D (7AAD; Beckman Coulter, Fullerton, CA) and the amount of p53 was determined by flow cytometry in a Cell Lab Quanta SC MPL flow cytometer. Nutlin-3a increased p53 amount in the control sample but not in p53 siRNA-transfected cells. D) Immunoblots on whole cell-lysates from scrambled (control) or p53 siRNA-transfected cells probed for the Akt phosphorylation status. (TIF) [file pone.0029541.s002.tif]
